# Effects of an increase in emergency cases with difficulties in transport to hospital during the COVID‐19 pandemic on postoperative short‐term outcomes of colorectal perforation: A study based on the National Clinical Database

**DOI:** 10.1002/ags3.12887

**Published:** 2024-11-27

**Authors:** Shimpei Ogawa, Hideki Endo, Masahiro Yoshida, Tomomitsu Tsuru, Michio Itabashi, Hiroyuki Yamamoto, Yoshihiro Kakeji, Hideki Ueno, Ken Shirabe, Taizo Hibi, Akinobu Taketomi, Norihiko Ikeda, Masaki Mori

**Affiliations:** ^1^ The Japanese Society for Abdominal Emergency Medicine Tokyo Japan; ^2^ Department of Surgery, Institute of Gastroenterology Tokyo Women's Medical University Tokyo Japan; ^3^ Department of Healthcare Quality Assessment, Graduate School of Medicine The University of Tokyo Tokyo Japan; ^4^ Department of HBP and Gastrointestinal Surgery, International University of Health and Welfare School of Medicine Ichikawa Japan; ^5^ Department of Medical Education and Training Shin‐Koga Hospital Fukuoka Japan; ^6^ Database Committee The Japanese Society of Gastroenterological Surgery Tokyo Japan; ^7^ Division of Gastrointestinal Surgery, Department of Surgery Kobe University Graduate School of Medicine Kobe Japan; ^8^ Department of Surgery National Defense Medical College Saitama Japan; ^9^ The Japanese Society of Gastroenterological Surgery Tokyo Japan; ^10^ Division of Hepatobiliary and Pancreatic Surgery, Department of General Surgical Science, Graduate School of Medicine Gunma University Maebashi Japan; ^11^ The Japan Surgical Society Tokyo Japan; ^12^ Department of Pediatric Surgery and Transplantation Kumamoto University Graduate School of Medical Sciences Kumamoto Japan; ^13^ Department of Gastroenterological Surgery I Hokkaido University Graduate School of Medicine Sapporo Japan; ^14^ Department of Surgery Tokyo Medical University Tokyo Japan; ^15^ Tokai University School of Medicine Kanagawa Japan

**Keywords:** colorectal perforation, COVID‐19, emergency cases with difficulties in transport to hospital, National Clinical Database, postoperative short‐term outcomes

## Abstract

**Aim:**

During the COVID‐19 pandemic, there were delays in transport of emergency cases to hospital by ambulance due to increased difficulties in obtaining hospital acceptance. The aim of this study was to examine if this had a negative effect on postoperative short‐term outcomes in patients with colorectal perforation.

**Methods:**

The National Clinical Database (NCD) includes >95% of surgical cases in Japan. Postoperative 30‐day mortality, surgical mortality, and postoperative complications (Clavien–Dindo grade ≥3) were examined in 17 770 cases of colorectal perforation registered from 2019 to 2022 in the NCD. These outcomes were compared for cases with new COVID‐19 infection and emergency cases with difficulties in transport to hospital. Months were considered to have significantly high or low mortality or complication rates, if the 95% confidence interval (CI) of the standardized mortality (morbidity) ratio (SMR) did not contain 1.

**Results:**

Postoperative 30‐day mortality occurred in 1826 cases (10.3%), surgical mortality in 2382 cases (13.4%), and postoperative complications in 5276 cases (29.7%). Significantly higher SMRs were found for 30‐day mortality in November 2020 (1.44 [95% CI: 1.07–1.89]) and February 2021 (1.54 [95% CI: 1.14–2.03]), and for postoperative complications in June 2020 (1.27 [95% CI: 1.07–1.50]). In 2022, there were marked increases in new COVID‐19 cases and in emergency cases with difficulties in transport to hospital, but no month had a significantly high SMR.

**Conclusions:**

Emergency cases with difficulties in transport markedly increased during the COVID‐19 pandemic but had little effect on short‐term outcomes of colorectal perforation.

## Graphical abstract

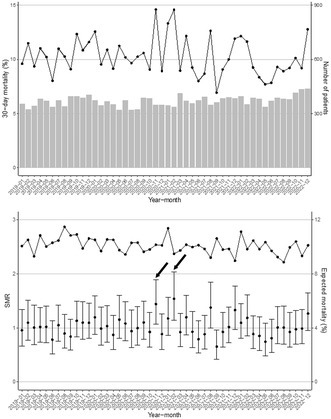

## 1 INTRODUCTION

The World Health Organization (WHO) announced the end of the public health emergency of international concern (PHEIC) for coronavirus disease 2019 (COVID‐19) on May 5, 2023.^1^ COVID‐19 originated in Wuhan, China and spread worldwide in 2019.^2^ More than 775 million people were infected with the disease and there were more than 7.05 million COVID‐19‐related deaths as of 3rd April 2024.^3^

The COVID‐19 pandemic had many impacts on the medical field. In the early period of the pandemic, less urgent medical treatment other than COVID‐19 was withheld and non‐emergency surgery was discontinued or delayed.^4^ The US Government requested postponement of hundreds of thousands of surgeries to reallocate hospital resources. The American College of Surgeons, Centers for Medicare and Medicaid Services, and other national societies published guidelines, including suggestion of postponement of nonessential services. Consequently, surgeries decreased by 50% between March and May 2020.^5^ In 190 United Nations member countries, approximately 28.4 million surgeries have been discontinued or delayed for 12 weeks since March 2020; that is, since the early pandemic period.^6^

A decrease in transport of emergency cases to hospital and prolonged transfer times may have delayed therapeutic interventions and resulted in poor outcomes.^7^, ^8^, ^9^ A report from France found significantly higher 90‐day mortality during and after the Nationwide Containment Rules by Emergency Declaration, compared to that before this period, in patients with sigmoid colonic diverticula, a common cause of intestinal perforation, treated with surgery and in patients who were complicated with peritonitis.^10^

In a previous study, we examined the short‐term outcomes of patients in Japan after surgery for colorectal perforation before and during the COVID‐19 pandemic.^11^ The results showed no clear negative effect of the COVID‐19 pandemic on these outcomes. However, the subjects were patients treated until December 2021. In 2022, newly infected COVID‐19 cases and emergency cases with difficulties in transport to hospital markedly increased in Japan.^12^, ^13^ Colorectal perforation has a poor prognosis that is accompanied by peritonitis and causes severe complications and sepsis in cases with delayed diagnosis and therapeutic intervention. Thus, a prolonged transport time may have adverse effects on outcomes.

Several studies have examined the impact of delayed transport of emergency cases with difficulties in transport on therapeutic outcomes,^14^, ^15^, ^16^, ^17^ but none have considered the effects on outcomes after surgery for colorectal perforation. Thus, this is the first study in Japan to examine whether increased emergency cases with difficulties in transport due to the COVID‐19 pandemic had a negative effect on postoperative short‐term outcomes of colorectal perforation.

## 2 METHODS

### 2.1 Patients

The subjects were patients with colorectal perforation who underwent surgery from 1st January to 31st December, 2022 and subjects of a previous study (i.e., patients with colorectal perforation who underwent surgery for acute diffuse peritonitis (ADP) from 1st January, 2019 to 31st December, 2021). All subjects were registered in the National Clinical Database (NCD). Patients with colorectal perforation had gastrointestinal perforation in the right‐sided colon (cecum and ascending colon), transverse colon, left‐sided colon (descending colon and sigmoid colon), and rectum, as a cause of peritonitis at this site. Of patients who underwent surgery for ADP, those aged less than 18 years or those with elective surgery, a missing value in observation items, or perforation at a site other than the colon were excluded from the study. The NCD was established in 2010 by clinical surgery societies supporting the system of specialists in Japan. The NCD now contains >95% of surgery cases in Japan, and more than 1.5 million cases have been registered annually since 2016.^18^

The study was approved by the ethics committee of Tokyo Women's Medical University (approval No. 2023‐0046) and the Japanese Society for Abdominal Emergency Medicine (approval No. 24‐02). This study was supported by the Ministry of Health, Labour and Welfare (MHLW) Research Program on Emerging and Re‐emerging Infectious Diseases and Immunization (Program Grant Number JPMH23HA2011 and JPMH24HA2015).

### 2.2 Outcomes

The effects of the increase in COVID‐19 cases and emergency cases with difficulties in transport to hospital on postoperative short‐term outcomes for colorectal perforation of 30‐day mortality, surgical mortality, and complications of Clavien–Dindo (CD) class ≥3 were compared in newly infected cases and emergency cases with difficulties in transport to hospital. Surgical mortality was defined as overall 30‐day mortality or in‐hospital death within 90 days. The number of newly infected patients with COVID‐19 was defined as that of new positive cases published by the MHLW.^12^ Emergency cases with difficulties in transport to hospital were defined as those needing four or more inquiries to health facilities by emergency service staff and detainment on site for at least 30 min, using data published by the Fire and Disaster Management Agency, including the Tokyo Fire Department and those in prefectural and ordinance designated city (more than 0.5 million population) governments (52 departments in total).^13^

### 2.3 Statistical analysis

The study was conducted using the standardized mortality (morbidity) ratio (SMR): SMR = observed mortality (morbidity)/expected mortality (morbidity). Logistic regression was used to estimate the expected mortality (morbidity) using factors associated with acute diffuse peritonitis reported in previous studies (age, sex, body mass index [BMI], American Society of Anesthesiologists [ASA] score, performance status [PS], activities of daily living [ADL], sepsis, malignant tumor, acute renal failure, hypertension, chronic obstructive pulmonary disease [COPD], bleeding risk, long‐term steroids, smoking, drinking, diabetes, leukocyte count, hemoglobin, platelet count, C‐reactive protein [CRP], total bilirubin, creatinine, prothrombin time with an international normalized ratio [PT‐INR], activated partial thromboplastin time [APTT], laparoscopy, and emergency transport).^19^ Months were considered to have significantly high or low mortality (morbidity), if the 95% confidence interval of the SMR does not contain 1. Statistical significance was defined as *p* < 0.05 in a *t*‐test. Statistical analysis was performed using R ver. 4.1.2 (2021; R Foundation for Statistical Computing, Vienna, Austria).

## 3 RESULTS

### 3.1 Patient characteristics

**FIGURE 1:**
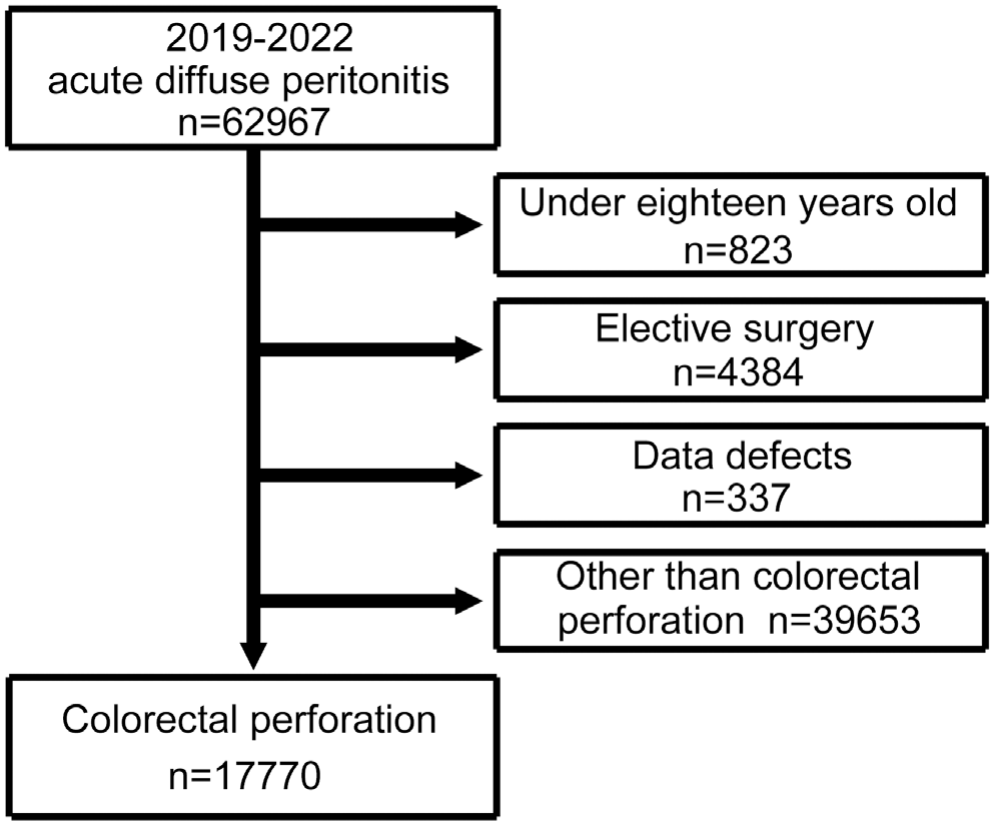
Flowchart of the patient selection process.

Of 62 967 patients with ADP who underwent surgery and were registered in the NCD between January 1st, 2019 and December 31st, 2022, 17 770 with colorectal perforation were included in the study, after excluding 823 patients aged <18 years, 4384 with elective surgery, 337 with a missing value in observation items, and 39 653 without colorectal perforation (Figure 1). Patient background factors are shown in Table 1.

**TABLE 1:** Characteristics of patients.

	Overall	2019	2020	2021	2022
*n*	17 770	4406	4242	4459	4663
Age (years) (%)					
<59	2986 (16.8)	691 (15.7)	753 (17.8)	731 (16.4)	811 (17.4)
≥60 to <64	1313 (7.4)	327 (7.4)	325 (7.7)	315 (7.1)	346 (7.4)
≥65 to <69	1905 (10.7)	518 (11.8)	459 (10.8)	506 (11.3)	422 (9.0)
≥70 to <74	2873 (16.2)	660 (15.0)	647 (15.3)	778 (17.4)	788 (16.9)
≥75 to <79	2664 (15.0)	716 (16.3)	623 (14.7)	627 (14.1)	698 (15.0)
≥80	6029 (33.9)	1494 (33.9)	1435 (33.8)	1502 (33.7)	1598 (34.3)
Sex (%)					
Male	9377 (52.8)	2332 (52.9)	2198 (51.8)	2371 (53.2)	2476 (53.1)
Female	8393 (47.2)	2074 (47.1)	2044 (48.2)	2088 (46.8)	2187 (46.9)
Body mass index (BMI) (%)					
<18.5 kg/m^2^	3802 (21.4)	985 (22.4)	908 (21.4)	925 (20.7)	984 (21.1)
≥18.5 to <25 kg/m^2^	10 566 (59.5)	2595 (58.9)	2500 (58.9)	2679 (60.1)	2792 (59.9)
≥25 kg/m^2^	3402 (19.1)	826 (18.7)	834 (19.7)	855 (19.2)	887 (19.0)
ASA PS (%)					
ASA1	787 (4.4)	211 (4.8)	193 (4.5)	192 (4.3)	191 (4.1)
ASA2	6522 (36.7)	1620 (36.8)	1563 (36.8)	1669 (37.4)	1670 (35.8)
ASA3–5	10 461 (58.9)	2575 (58.4)	2486 (58.6)	2598 (58.3)	2802 (60.1)
Independence in ADL (%)					
+	5080 (28.6)	1241 (28.2)	1176 (27.7)	1287 (28.9)	1376 (29.5)
Sepsis (%)					
None	14 039 (79.0)	3351 (76.1)	3366 (79.3)	3562 (79.9)	3760 (80.6)
Sepsis	1775 (10.0)	501 (11.4)	421 (9.9)	407 (9.1)	446 (9.6)
Septic shock	1956 (11.0)	554 (12.6)	455 (10.7)	490 (11.0)	457 (9.8)
Malignancy (%)					
+	3808 (21.4)	930 (21.1)	900 (21.2)	907 (20.3)	1071 (23.0)
Acute renal failure (%)					
+	523 (2.9)	139 (3.2)	122 (2.9)	132 (3.0)	130 (2.8)
Hypertension (%)					
+	7939 (44.7)	1921 (43.6)	1867 (44.0)	2000 (44.9)	2151 (46.1)
COPD (%)					
+	585 (3.3)	161 (3.7)	150 (3.5)	119 (2.7)	155 (3.3)
Risk of hemorrhage (%)					
+	2018 (11.4)	484 (11.0)	503 (11.9)	525 (11.8)	506 (10.9)
Long‐term steroid use (%)					
+	1214 (6.8)	300 (6.8)	286 (6.7)	301 (6.8)	327 (7.0)
Smoking (%)					
+	2925 (16.5)	734 (16.7)	713 (16.8)	733 (16.4)	745 (16.0)
Habitual alcohol consumption (%)					
+	4024 (22.6)	965 (21.9)	935 (22.0)	1036 (23.2)	1088 (23.3)
Diabetes mellitus (%)					
+	2735 (15.4)	677 (15.4)	658 (15.5)	682 (15.3)	718 (15.4)
WBC count (%)					
<3500/μl	3172 (17.9)	790 (17.9)	775 (18.3)	788 (17.7)	819 (17.6)
≥3500 to <9000/μl	6664 (37.5)	1683 (38.2)	1589 (37.5)	1692 (37.9)	1700 (36.5)
≥9000/μl	7899 (44.5)	1925 (43.7)	1870 (44.1)	1972 (44.2)	2132 (45.7)
Unknown	35 (0.2)	8 (0.2)	8 (0.2)	7 (0.2)	12 (0.3)
Hemoglobin (%)					
M: <13.5 g/dL, F: <11.5 g/dL	9498 (53.4)	2359 (53.5)	2227 (52.5)	2370 (53.2)	2542 (54.5)
M: ≥13.5 g/dL to ≤17.0 g/dL F ≥ 11.5 g/dL to ≤15.0 g/dL	7639 (43.0)	1892 (42.9)	1876 (44.2)	1906 (42.7)	1965 (42.1)
M: >17.0 g/dL, F: 15 g/dL	580 (3.3)	136 (3.1)	130 (3.1)	172 (3.9)	142 (3.0)
Unknown	53 (0.3)	19 (0.4)	9 (0.2)	11 (0.2)	14 (0.3)
Platelets (%)					
<150 000	2576 (14.5)	667 (15.1)	624 (14.7)	648 (14.5)	637 (13.7)
≥150 000 to ≤350 000	12 327 (69.4)	3061 (69.5)	2961 (69.8)	3094 (69.4)	3211 (68.9)
>350 000	2825 (15.9)	668 (15.2)	648 (15.3)	710 (15.9)	799 (17.1)
Unknown	42 (0.2)	10 (0.2)	9 (0.2)	7 (0.2)	16 (0.3)
C‐reactive protein (CRP) (%)					
≤0.1 mg/dL	2101 (11.8)	564 (12.8)	497 (11.7)	559 (12.5)	481 (10.3)
>0.1 mg/dL to ≤1.0 mg/dL	2359 (13.3)	590 (13.4)	590 (13.9)	609 (13.7)	570 (12.2)
>1.0 mg/dL to ≤5.0 mg/dL	2446 (13.8)	607 (13.8)	561 (13.2)	610 (13.7)	668 (14.3)
>5.0 mg/dL to ≤10.0 mg/dL	1896 (10.7)	472 (10.7)	452 (10.7)	469 (10.5)	503 (10.8)
>10.0 mg/dL	8652 (48.7)	2075 (47.1)	2067 (48.7)	2140 (48.0)	2370 (50.8)
Unknown	316 (1.8)	98 (2.2)	75 (1.8)	72 (1.6)	71 (1.5)
Total bilirubin (%)					
≤1.0 mg/dL	14 395 (81.0)	3547 (80.5)	3427 (80.8)	3581 (80.3)	3840 (82.4)
>1.0 mg/dL	3224 (18.1)	821 (18.6)	776 (18.3)	843 (18.9)	784 (16.8)
Unknown	151 (0.8)	38 (0.9)	39 (0.9)	35 (0.8)	39 (0.8)
Creatinine (%)					
≤1.0 mg/dL	10 307 (58.0)	2612 (59.3)	2508 (59.1)	2579 (57.8)	2608 (55.9)
>1.0 mg/dL	7387 (41.6)	1774 (40.3)	1719 (40.5)	1858 (41.7)	2036 (43.7)
Unknown	76 (0.4)	20 (0.5)	15 (0.4)	22 (0.5)	19 (0.4)
PT‐INR (%)					
<0.9	427 (2.4)	92 (2.1)	79 (1.9)	114 (2.6)	142 (3.0)
≥0.9 to ≤1.1	9844 (55.4)	2437 (55.3)	2314 (54.5)	2475 (55.5)	2618 (56.1)
>1.1	6543 (36.8)	1607 (36.5)	1623 (38.3)	1629 (36.5)	1684 (36.1)
Unknown	956 (5.4)	270 (6.1)	226 (5.3)	241 (5.4)	219 (4.7)
APTT (%)					
<30 s	5226 (29.4)	1245 (28.3)	1223 (28.8)	1332 (29.9)	1426 (30.6)
≥30 to ≤40 s	9128 (51.4)	2268 (51.5)	2210 (52.1)	2276 (51.0)	2374 (50.9)
>40 s	2348 (13.2)	600 (13.6)	550 (13.0)	584 (13.1)	614 (13.2)
Unknown	1068 (6.0)	293 (6.7)	259 (6.1)	267 (6.0)	249 (5.3)
Laparoscopic surgery (%)					
+	2080 (11.7)	438 (9.9)	500 (11.8)	572 (12.8)	570 (12.2)
Ambulance transportation (%)					
+	8878 (50.0)	2116 (48.0)	2067 (48.7)	2263 (50.8)	2432 (52.2)
30‐day mortality (%)	1826 (10.3)	460 (10.4)	448 (10.6)	463 (10.4)	455 (9.8)
Surgical mortality (%)	2382 (13.4)	615 (14.0)	587 (13.8)	603 (13.5)	577 (12.4)
Complications (CD ≥3) (%)	5276 (29.7)	1364 (31.0)	1309 (30.9)	1277 (28.6)	1326 (28.4)
Postoperative hospital stays, days (median [IQR])	26 [16, 46]	28 [17, 49]	26 [16, 45]	26 [16, 44]	26 [16, 44]

Abbreviations: ADL, activities of daily living; APTT: activated partial thromboplastin time; ASA PS, American Society of Anesthesiologists physical status; CD, Clavien–Dindo classification grade; COPD, chronic obstructive pulmonary disease; IQR, interquartile range; PT‐INR, prothrombin time‐international normalized ratio; WBC, white blood cell.

### 3.2 Difficulty obtaining hospital acceptance for transfer of a patient

**FIGURE 2:**
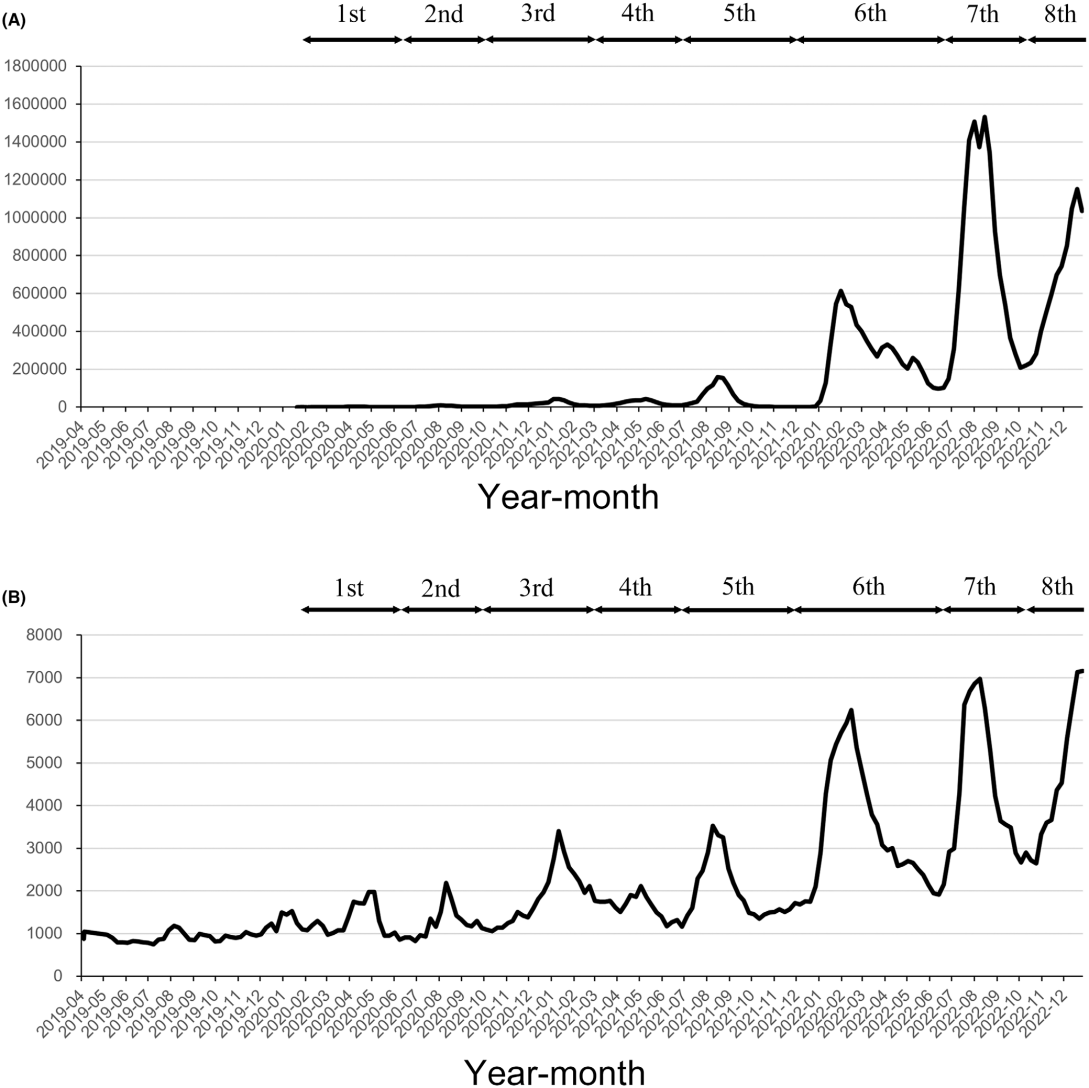
(A) New COVID‐19 patient. (Data [Information on infectious disease outbreak trends, etc.] from the website has been quoted and modified [Ministry of Health, Labour and Welfare, Japan. https://covid19.mhlw.go.jp/public/aboutdata.html. Accessed 15 March 2024]); (B) Emergency case with difficulties in transport to hospital. (Data [Results of a survey on difficult emergency transport cases from each fire department.] from the website has been quoted and modified [Fire and Disaster Management Agency. Survey on the difficulty in hospital acceptance during the novel corona virus pandemic. https://www.fdma.go.jp/disaster/coronavirus/post‐1.html. Accessed 15 March 2024]). First wave: weeks 3–23 in 2020 (13 Jan–7 Jun 2020). Second wave: weeks 24–39 in 2020 (8 Jun–27 Sep 2020). Third wave: week 40 in 2020 to week 8 in 2021 (28 Sep 2020–28 Feb 2021). Fourth wave: weeks 9–24 in 2021 (29 Feb–20 Jun 2021). Fifth wave: weeks 25–47 in 2021 (21 Jun–28 Nov 2021). Sixth wave: week 48 in 2021 to week 24 in 2022 (29 Nov 2021–19 Jun 2022). Seventh wave: weeks 25–40 in 2022 (20 Jun–9 Oct 2022). Eighth wave: week 41 in 2022 to week 13 in 2023 (10 Oct 2022–2 May 2023).

Eight pandemic waves of COVID‐19 occurred from 2020 to 2022. The start of a pandemic wave was defined as the third week in which COVID‐19 cases increased continuously.^20^ The number of emergency cases with difficulties in transport to hospital changed in parallel with the number of newly infected patients. Especially in 2022, newly infected patients increased markedly in the sixth, seventh, and eighth pandemic waves and emergency cases with difficulties with transport also increased. The highest number of patients was 6237 in epidemiological week (hereinafter referred to as week) 7 during the sixth pandemic wave and 6975 in week 32 during the seventh pandemic wave. During the eighth pandemic wave, the highest numbers of patients was 8322 in week 2 in 2023, while the highest number of patients was 7155 in week 52 (Figure 2).

### 3.3 SMR (Figure 3)

**FIGURE 3:**
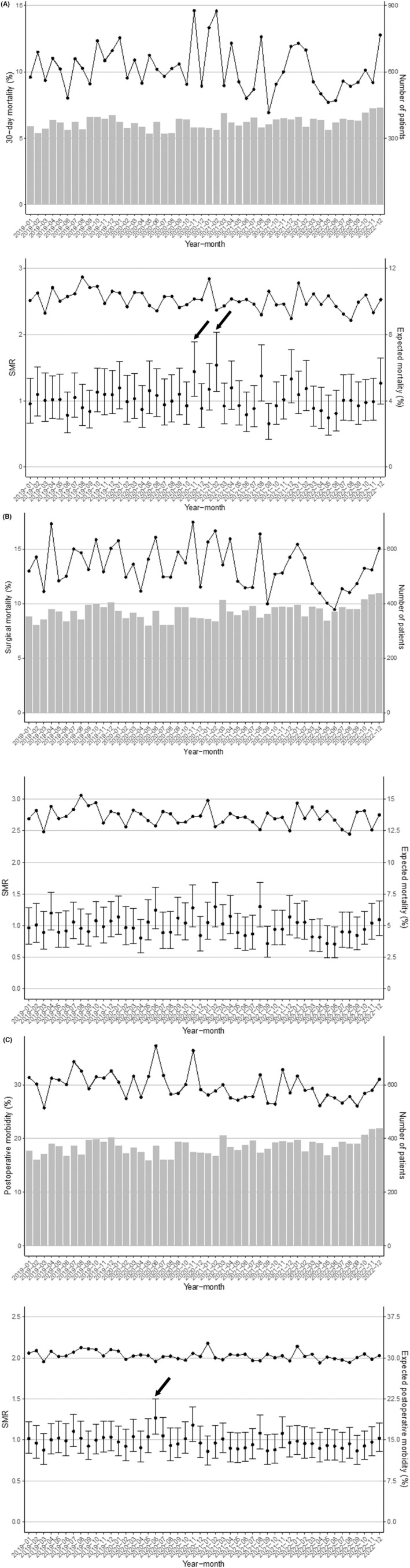
Standardized mortality (morbidity) ratio (SMR) by month for all subjects. (A) Top: 30‐day mortality and number of patients. Bottom: SMR and expected mortality. (B) Top: surgical mortality and number of patients. Bottom: SMR and expected mortality. (C) Top: postoperative complications (Clavien–Dindo classification grade ≥3) and number of patients. Bottom: SMR and expected morbidity. → indicates months when the SMR was significantly higher.

The postoperative 30‐day mortality was 1826 (10.3%) in the study period. A significantly high SMR for 30‐day mortality was found in November 2020 (1.44 [95% CI: 1.07–1.89]) and February 2021 (1.54 [95% CI: 1.14–2.03]), while SMR was significantly low in September 2021 (0.65 [95% CI: 0.42–0.96]). The surgical mortality was 2382 (13.4%). No month had a significantly high SMR for surgical mortality, but SMR was significantly low in September 2021 (0.72 [95% CI: 0.50–0.99]) and June 2022 (0.71 [95% CI: 0.49–0.99]). There were 5276 postoperative complications (CD ≥3) (29.7%), with a significantly high SMR in June 2020 (1.27 [95% CI: 1.07–1.50]).

June 2020 was in the first and second waves, and November 2020 and February 2021 were in the third wave; however, these periods did not correspond to peaks of emergency cases with difficulties in transport. In addition, new COVID‐19 patients in 2022 greatly increased in the sixth, seventh, and eighth pandemic waves and emergency cases with difficulties in transport increased until the fifth pandemic wave. However, there was no significantly high SMR in any of the corresponding months (Appendix S1).

## 4 DISCUSSION

Significantly high SMRs were found for 30‐day mortality in November 2020 and February 2021, and for complications (CD ≥3) in June 2020, with none found for surgical mortality. There were increased emergency cases with difficulties in transport to hospital during the COVID‐19 pandemic, especially in 2022, when there was a particularly marked increase in newly infected cases and emergency cases with difficulties in transport to hospital. However, SMR was not significantly high in any month in 2022. Thus, emergency cases with difficulties in transport to hospital increased due to the COVID‐19 pandemic, but there was little effect on postoperative short‐term outcomes of colorectal perforation.

An emergency case with difficulties in transport to hospital was defined as a case that needed four or more inquiries to health facilities by emergency service staff and remained on site for at least 30 min.^13^ The factors for difficulty in acceptance of emergency cases include elderly patients, foreign patients, and nights, weekends, and holidays.^21^ There have been reports of increased emergency cases with difficulties in transport to hospital during the COVID‐19 pandemic.^17^, ^22^ The Osaka Government reported that of emergency cases registered in the Osaka Emergency Information Research Intelligent Operation Network (ORION) system, 2.74% were emergency cases with difficulties in transport to hospital in 2019, 3.74% in 2020, and 5.09% in 2021.^17^ The study from Tokyo also reported a higher rate of emergency cases with difficulties in transport to hospital in 2020 than in 2019.^22^ This study also showed a positive correlation of new COVID 19 cases with emergency cases with difficulties in transport to hospital and field activity time; that is, emergency cases with difficulties in transport to hospital increased by 86.4 cases and field activity time was prolonged by 3.48 min/1000 newly infected cases per day. Similarly, a study in Korea found that the response time interval, scene time interval, and transport time interval for emergency transport times were shorter in 2017 to 2019 than in 2020 to 2022.^23^ The mean turnaround time intervals were 27.6, 27.9, and 28.7 min in 2017, 2018, and 2019, and 35.2, 42.0, and 43.1 min in 2020, 2021, and 2022, respectively, suggesting prolongation in 2020 and thereafter.

A possible cause of increased emergency cases with difficulties in transport to hospital is that patients were concentrated in certain medical facilities.^17^, ^22^ COVID‐19 cases, and especially severe patients, were treated in specific medical facilities with intensive care units.^24^ These facilities are core hospitals in which severe and urgent diseases are treated, including colorectal perforation. During the COVID‐19 pandemic, patients with fever and sore throat were treated in these facilities, and COVID‐19 infection could not be ruled out, even if the final diagnosis was another disease. Consequently, patients accumulated in these facilities and cases that had previously been treated there could not be accepted, resulting in increased emergency cases with difficulties in transport to hospital.

Several studies have suggested that increased emergency cases with difficulties in transport to hospital influenced outcomes during the COVID‐19 pandemic. A report in the United States indicated that transported emergency cases decreased from week 10 onwards in 2020 and that severer cases could not be treated in a timely manner, resulting in a doubling of EMS‐attended deaths in weeks 11–15.^14^ In Italy, that patients with out‐of‐hospital cardiac arrest (OHCA) in 2020 increased by 58% in comparison with those in 2019, and the cumulative incidence of OHCA in 2020 was highly correlated with that of COVID‐19.^15^ A study in Australia showed that survival rates of OHCA decreased by 50% due to increased emergency cases with difficulties in transport to hospital, prolonged transport times, and marked delays in critical therapeutic intervention.^16^

In Japan, the numbers of patients who died during emergency outpatient service in emergency cases with difficulties in transport to hospital registered in the ORION system were 13 in 2019, 19 in 2020, and 39 in 2021, while patients who died within 21 days after admission increased from 218 in 2019 to 405 in 2020 and 750 in 2021.^17^ The major causes of death were diseases of the respiratory or circulatory system; however, deaths of patients with diseases of the digestive system also increased. In addition to the increased emergency cases with difficulties in transport to hospital, the ability of medical staff to provide treatment was reduced due to infection control, and the frequency of first aid prior to hospital admission was also decreased. As a result, patients who needed urgent treatment could not be treated in a timely manner, resulting in a poor effect on outcomes.^17^, ^22^

Most patients with colorectal perforation complicate with peritonitis, and progression to diffuse peritonitis is likely to cause septic shock, which needs prompt therapeutic intervention.^25^, ^26^ In Japan, COVID‐19 cases increased markedly in 2022 compared to those until 2021. After the pandemic started, emergency cases with difficulties in transport to hospital increased, especially in the sixth, seventh, and eighth waves, which had major increases in newly infected patients. Therefore, delayed therapeutic intervention associated with increased emergency cases with difficulties in transport to hospital might have had negative effects on outcomes of patients who underwent surgery for colorectal perforation. However, the number of surgeries in 2022 was similar to that in previous years and SMR was not significantly high in any month in 2022.

There may be several reasons for the absence of a negative effect on short‐term outcomes of patients who underwent surgery for colorectal perforation during the pandemic. First, colorectal perforation requires emergency surgery in most patients and there is no alternative therapy; therefore, the number of surgeries did not change. In the Japan Surgical Society “Recommendations for surgery of COVID‐19‐positive/suspected patients,” colorectal perforation was classified in Disease Level C, for which procedures other than surgery should be chosen if possible, and patients who cannot be treated with such procedures should undergo emergency surgery.^27^ In addition to the effects of these recommendations, all surgery professionals committed to these decisions in tight situations, leading to appropriate surgery and perioperative control. This was also expected based on the so‐called “weekend effect” in the emergency medical system in Japan. A study of differences in therapeutic intervention between weekdays and weekends indicated that operative mortality in ADP cases with colorectal perforation did not differ by day of the week, and that the prognosis was not worse for patients admitted on the weekend. This suggests that the quality of surgery for ADP remained high at night and at weekends.^28^

A second reason may be the urgency of therapeutic intervention, which depends on the disease. Colorectal perforation requires emergency surgery, but all patients do not complicate with diffuse peritonitis; some have local peritonitis and there is time to wait until therapeutic intervention. It has been reported in Japan that increased emergency cases with difficulties in transport to hospital influenced treatment outcomes in emergency departments, but the major effects were for diseases of the respiratory and circulatory system.^17^ These diseases are most likely to become serious over a short time and require prompt attention. Colorectal perforation is relatively less affected because therapeutic intervention can be somewhat delayed in some cases.

In the early COVID‐19 pandemic, infectivity, pathology, and treatment of COVID‐19 derived from an unknown virus were not fully understood, and medical facilities had to manage patients by trial and error. Infection control measures were improved and test systems were established, and then vaccination began to control COVID‐19, leading to improved management.^29^ Since 2022, there was a major increase in newly infected patients and emergency cases with difficulties in transport to hospital. However, negative effects were less than in the early COVID‐19 pandemic. The study from Tokyo reported that the number of emergency cases with difficulties in transport to hospital were 516, 154, and 97 per 1000 newly infected patients per day and the extended activity times on the scene were 25.6, 3.61, and 4.08 min in the first, second, and third waves.^22^

In this study, significantly high SMRs were found in June and November 2020 and February 2021, but none thereafter. SMRs in September 2021 and June 2022 were significantly low, which may suggest the recovery of medical systems. The effect of the COVID‐19 pandemic on increased emergency cases with difficulties in transport to hospital was due to concentration of patients in certain medical facilities. Appropriate personnel distribution in medical facilities was required to secure facility resources during the pandemic. In emergency departments, there was a need to understand that the urgency of therapeutic intervention depends on the disease state. Thus, appropriate triage, including selection of patients requiring priority for prompt intervention, is a measure for consideration for a future pandemic.

This study has the following limitations. The data were taken from the number of emergency cases with difficulties in transport to hospital published by the Fire and Disaster Management Agency, including the Tokyo Fire Department and those in prefectural and ordinance designated city governments (52 departments in total).^13^ The changes reveal the current status in Japan; however, emergency cases with difficulties in transport to hospital in other fire and disaster management areas are not included, and consequently, the number of emergency cases with difficulties in transport to hospital does not reflect the number throughout Japan.

Surgical cases of colorectal perforation include many patients whose general condition is relatively well maintained, but a certain number of patients with a poor general condition are also included. Due to the high possibility of undesirable outcomes in many patients with a poor general condition who do not undergo surgery, a surgical procedure is often performed for such patients in clinical practice after a sufficient explanation is given to the patients and their families. A complete understanding of the current situation requires consideration of individual preoperative general conditions, the number of patients who could not undergo surgery due to a poor general condition, and the presence of patients who could not receive surgery due to restricted medical services.

Various factors including perforation sites, contamination status in the abdominal cavity, and the general condition of the patient are involved in treatment outcomes of colorectal perforation. In this study, risks were adjusted by excluding confounding factors as much as possible and examined using SMR. However, NCD data do not include the time from onset to treatment intervention, or the capacity and function of facilities in which treatment was performed. The analysis of facilities throughout Japan did not show an effect on treatment outcomes; however, the results do not necessarily indicate that there was no impact at each facility. The capacity and function of the facility may influence treatment outcomes, and there is a need to analyze the data by facility with consideration of these conditions.

## 5 CONCLUSION

The NCD includes most surgery cases in Japan and, thus, provides a basis for examining postoperative short‐term outcomes in patients with colorectal perforation before and during the COVID‐19 pandemic. In 2022, emergency cases with difficulties in transport markedly increased during the COVID‐19 pandemic, but had little effect on short‐term outcomes of colorectal perforation. These data suggest that the emergency system for patients with colorectal perforation was largely maintained during the pandemic in Japan, with no evidence indicating a serious breakdown of the system.

## AUTHOR CONTRIBUTIONS

**Shimpei Ogawa:** Conceptualization; formal analysis; investigation; methodology; project administration; writing – original draft. **Hideki Endo:** Conceptualization; data curation; formal analysis; investigation; methodology; project administration; writing – review and editing. **Masahiro Yoshida:** Conceptualization; formal analysis; project administration; writing – review and editing. **Tomomitsu Tsuru:** Conceptualization; formal analysis; project administration; writing – review and editing. **Michio Itabashi:** Conceptualization; formal analysis; project administration; writing – review and editing. **Hiroyuki Yamamoto:** Conceptualization; data curation; formal analysis; investigation; methodology; project administration; writing – review and editing. **Yoshihiro Kakeji:** Conceptualization; formal analysis; project administration; writing – review and editing. **Hideki Ueno:** Conceptualization; formal analysis; project administration; writing – review and editing. **Ken Shirabe:** Conceptualization; formal analysis; project administration; writing – review and editing. **Taizo Hibi:** Conceptualization; formal analysis; project administration; writing – review and editing. **Akinobu Taketomi:** Conceptualization; formal analysis; project administration; writing – review and editing. **Norihiko Ikeda:** Conceptualization; formal analysis; project administration; writing – review and editing. **Masaki Mori:** Conceptualization; formal analysis; project administration; writing – review and editing.

## FUNDING INFORMATION

This work was supported by MHLW Research on Emerging and Re‐emerging Infectious Diseases and Immunization (Program Grant Number JPMH23HA2011 and JPMH24HA2015).

## CONFLICT OF INTEREST STATEMENT

Hideki Endo and Hiroyuki Yamamoto are affiliated with the Department of Healthcare Quality Assessment at the University of Tokyo. The department is a social collaboration department supported by grants from the National Clinical Database, Intuitive Surgical Sarl, Johnson & Johnson K.K., and Nipro Co. Masaki Mori is Emeritus Editor‐in‐Chief of *Annals of Gastroenterological Surgery*. Hideki Ueno is Associate Editor of *Annals of Gastroenterological Surgery* dealing with the lower digestive tract. Yoshihiro Kakeji and Ken Shirabe are editorial members of *Annals of Gastroenterological Surgery*. The other authors declare no conflict of interest for this article.

## ETHICS STATEMENT

Approval of the research protocol by an Institutional Reviewer Board: This study was approved by the ethics committee of Tokyo Women's Medical University (approval no. 2023‐0046) and the Japanese Society for Abdominal Emergency Medicine (approval no. 24‐02).

Informed Consent: Patients agreed to inclusion of their data in this study through presumed consent with opt‐out through a web page and/or a notice from each center.

Registry and the Registration No. of the study/trial: N/A.

Animal Studies: N/A.

## Supporting information


Appendix S1.
