# Supplementary material for: Effects of an increase in emergency cases with difficulties in transport to hospital during the COVID‐19 pandemic on postoperative short‐term outcomes of colorectal perforation: A study based on the National Clinical Database
Source: Ann Gastroenterol Surg. 2024 Nov 27;9(3):505–17. doi: 10.1002/ags3.12887 (PMC12080204; doi:10.1002/ags3.12887)
Supplement: Supplementary file 1 — Appendix S1. [file AGS3-9-505-s001.docx]

Supplement 1. Short-term outcomes for colorectal perforation

| 2019 |  | 2019.1 | 2019.2 | 2019.3 | 2019.4 | 2019.5 | 2019.6 | 2019.7 | 2019.8 | 2019.9 | 2019.10 | 2019.11 | 2019.12 |
| --- | --- | --- | --- | --- | --- | --- | --- | --- | --- | --- | --- | --- | --- |
| n | 4406 | 354 | 322 | 342 | 381 | 372 | 336 | 373 | 341 | 396 | 397 | 387 | 405 |
| 30-day mortality (%) | 460  (10.4) | 34 (9.6) | 37 (11.5) | 32 (9.4) | 42 (11.0) | 38 (10.2) | 27 (8.0) | 41 (11.0) | 35 (10.3) | 36 (9.1) | 49 (12.3) | 42 (10.9) | 47 (11.6) |
| Surgical mortality (%) | 615  (14.0) | 46 (13.0) | 46 (14.3) | 38 (11.1) | 66 (17.3) | 45 (12.1) | 42 (12.5) | 56 (15.0) | 50 (14.7) | 52 (13.1) | 63 (15.9) | 50 (12.9) | 61 (15.1) |
| Complications (CD≥3) (%) | 1364  (31.0) | 111 (31.4) | 97 (30.1) | 88 (25.7) | 119 (31.2) | 115 (30.9) | 101 (30.1) | 128 (34.3) | 111 (32.6) | 116 (29.3) | 125 (31.5) | 121 (31.3) | 132 (32.6) |
| Postoperative hospital stay, days (median [IQR]) | 28 [17, 49] | 30 [17, 48] | 28 [18, 50] | 29 [18, 52] | 30 [16, 48] | 28 [17, 54] | 29 [17, 50] | 26 [17, 47] | 29 [18, 51] | 26 [17, 44] | 25 [15, 45] | 26 [16, 49] | 28 [16, 47] |
|  |  |  |  |  |  |  |  |  |  |  |  |  |  |
| 2020 |  | 2020.1 | 2020.2 | 2020.3 | 2020.4 | 2020.5 | 2020.6 | 2020.7 | 2020.8 | 2020.9 | 2020.10 | 2020.11 | 2020.12 |
| n | 4242 | 374 | 346 | 367 | 350 | 320 | 373 | 321 | 322 | 387 | 386 | 349 | 347 |
| 30-day mortality (%) | 448  (10.6) | 47 (12.6) | 33 (9.5) | 40 (10.9) | 32 (9.1) | 36 (11.2) | 38 (10.2) | 31 (9.7) | 33 (10.2) | 41 (10.6) | 35  (9.1) | 51 (14.6) | 31  (8.9) |
| Surgical mortality (%) | 587  (13.8) | 59 (15.8) | 43 (12.4) | 50 (13.6) | 39 (11.1) | 45 (14.1) | 60 (16.1) | 40 (12.5) | 40 (12.4) | 57 (14.7) | 53 (13.7) | 61 (17.5) | 40 (11.5) |
| Complications (CD≥3) (%) | 1309  (30.9) | 114 (30.5) | 95 (27.5) | 116 (31.6) | 97 (27.7) | 101 (31.6) | 139 (37.3) | 102 (31.8) | 91 (28.3) | 110 (28.4) | 116 (30.1) | 127 (36.4) | 101 (29.1) |
| Postoperative hospital stay, days (median [IQR]) | 26 [16, 45] | 26 [16, 40] | 25 [16, 45] | 27 [17, 44] | 25 [15, 45] | 27 [16, 47] | 27 [16, 48] | 30 [17, 48] | 28 [17, 48] | 25 [16, 42] | 25 [16, 43] | 25 [15, 43] | 26 [16, 48] |
|  |  |  |  |  |  |  |  |  |  |  |  |  |  |
| 2021 |  | 2021.1 | 2021.2 | 2021.3 | 2021.4 | 2021.5 | 2021.6 | 2021.7 | 2021.8 | 2021.9 | 2021.10 | 2021.11 | 2021.12 |
| n | 4459 | 345 | 336 | 413 | 370 | 357 | 375 | 392 | 348 | 361 | 386 | 390 | 386 |
| 30-day mortality (%) | 463  (10.4) | 46 (13.3) | 49 (14.6) | 37 (9.0) | 45 (12.2) | 33 (9.2) | 30 (8.0) | 34 (8.7) | 44 (12.6) | 25 (6.9) | 35  (9.1) | 39 (10.0) | 46 (11.9) |
| Surgical mortality (%) | 603  (13.5) | 54 (15.7) | 56 (16.7) | 56 (13.6) | 59 (15.9) | 43 (12.0) | 43 (11.5) | 45 (11.5) | 57 (16.4) | 36 (10.0) | 49 (12.7) | 50 (12.8) | 55 (14.2) |
| Complications (CD≥3) (%) | 1277  (28.6) | 97 (28.1) | 97 (28.9) | 124 (30.0) | 102 (27.6) | 97 (27.2) | 104 (27.7) | 109 (27.8) | 111 (31.9) | 96 (26.6) | 102 (26.4) | 128 (32.8) | 110 (28.5) |
| Postoperative hospital stay, days (median [IQR]) | 26 [16, 44] | 25 [16, 43] | 26 [15, 43] | 26 [16, 41] | 24 [16, 42] | 27 [17, 42] | 27 [16, 49] | 26 [17, 47] | 26 [15, 44] | 26 [17, 41] | 25 [16, 49] | 27 [17, 48] | 24 [15, 44] |
|  |  |  |  |  |  |  |  |  |  |  |  |  |  |
| 2022 |  | 2022.1 | 2022.2 | 2022.3 | 2022.4 | 2022.5 | 2022.6 | 2022.7 | 2022.8 | 2022.9 | 2022.10 | 2022.11 | 2022.12 |
| n | 4663 | 395 | 352 | 389 | 383 | 338 | 370 | 387 | 381 | 380 | 415 | 435 | 438 |
| 30-day mortality (%) | 455  (9.8) | 48 (12.2) | 41 (11.6) | 36 (9.3) | 32 (8.4) | 26 (7.7) | 29 (7.8) | 36 (9.3) | 34 (8.9) | 35 (9.2) | 42 (10.1) | 40  (9.2) | 56 (12.8) |
| Surgical mortality (%) | 577  (12.4) | 61 (15.4) | 50 (14.2) | 46 (11.8) | 42 (11.0) | 34 (10.1) | 35 (9.5) | 44 (11.4) | 42 (11.0) | 45 (11.8) | 55 (13.3) | 57 (13.1) | 66 (15.1) |
| Complications (CD≥3) (%) | 1326  (28.4) | 125 (31.6) | 102 (29.0) | 114 (29.3) | 100 (26.1) | 95 (28.1) | 102 (27.6) | 103 (26.6) | 106 (27.8) | 99 (26.1) | 118 (28.4) | 126 (29.0) | 136 (31.1) |
| Postoperative hospital stay, days (median [IQR]) | 26 [16, 44] | 27 [16, 45] | 24 [14, 41] | 26 [17, 44] | 27 [17, 45] | 25 [17, 45] | 27 [17, 44] | 26 [16, 41] | 27 [17, 46] | 26 [16, 43] | 29 [18, 51] | 26 [16, 41] | 25 [15, 42] |

Abbreviations: CD, Clavien-Dindo grade; IQR, interquartile range.
